# Nanofiltration Performance of Poly(*p*-xylylene) Nanofilms with Imidazole Side Chains

**DOI:** 10.3390/polym15153309

**Published:** 2023-08-04

**Authors:** Satsuki Yoshida, Takeshi Shii, Yu Kitazawa, Manuela L. Kim, Eugenio H. Otal, Yoshiyuki Hattori, Mutsumi Kimura

**Affiliations:** 1 Department of Chemistry and Materials, Faculty of Textile Science and Technology, Shinshu University, Ueda 386-8567, Japanhattoriy@shinshu-u.ac.jp (Y.H.); 2 Research Initiative for Supra-Materials (RISM), Interdisciplinary Cluster for Cutting Edge Research (ICCER), Shinshu University, Ueda 386-8567, Japan; 3 Global Aqua Innovation Center, Shinshu University, Nagano 380-8553, Japan

**Keywords:** chemical vapor deposition, membrane, hybrid materials, water purification, metal–organic framework

## Abstract

Herein, we report the nanofiltration performance of poly(*p*-xylylene) thin films with imidazole side chains that were deposited onto commercial polyethersulfone ultrafiltration membranes using a chemical vapor deposition process. The resulting thin films with a few tens of nanometers exhibited water permeation under a pressure difference of 0.5 MPa and selectively rejected water-soluble organic dyes based on their molecular sizes. Additionally, thin flaky ZIF-L crystals (Zn(mim)_2_·(Hmim)_1/2_·(H_2_O)_3/2_) (Hmim = 2-methylimidazole) formed on the surface of imidazole-containing poly(*p*-xylylene) films, and the composite films demonstrated the ability to adsorb methylene blue molecules within the cavities of ZIF-L.

## 1. Introduction

Membrane processes have shown great promise in separation due to their high performance and low energy consumption [1,2,3]. Membranes have been developed using materials including polymers [4,5], amphiphiles [6,7], metal–organic frameworks [8,9], and carbons [10,11,12] to enable breakthroughs in molecular separation processes. Polymer-based membranes, in particular, rely on the surface characteristics and the inner free volume of the polymers to achieve optimal performance. Technological advancements have been made to control attributes of polymer-based membranes, such as high permeability, good selectivity, and membrane fouling [13,14]. However, achieving precise structural control of large-area and defect-free membranes remains challenging. In our previous work, we reported the nanofiltration properties of ultrathin films based on crosslinked poly(xylylene)s (PPXs) with polar substituents deposited using the chemical vapor deposition process (CVD) [15]. By tuning surface structure and internal free volume in PPX films, we can create desired permeable pathways within the condensed polymer chains formed through bottom-up polymerization of functionalized [2.2]paracyclophane on the surface of target substrates. 

Since the mid-1960s, PPXs have been prepared through surface polymerization of *p*-quinodimethanes generated via vacuum vapor pyrolysis of [2.2]paracyclophane derivatives (known as the Gorham process) [16,17]. PPXs have been widely utilized as coating layers due to their excellent conformity, uniform thickness, and impressive thermal/chemical stabilities [18]. During the CVD process, a [2.2]paracyclophane monomer undergoes cleavage into reactive *p*-quinodimethane precursors above 550 °C under vacuum. The generated precursors then polymerize to semi-crystalline PPXs on target surfaces, resulting in defect-free and uniform coating layers. This coating method enables the adsorption of gaseous monomers onto the target surfaces. The PPX coating films offer flexible layers that serve as barriers for gases and liquids, corrosion control agents, and lubricants on various surfaces [19,20]. Additionally, PPX films have become integral components in realizing stretchable electronics and bioactive surfaces [21]. Someya et al. successfully fabricated stretchable organic-based electronic devices, such as light-emitting diodes, photodetectors, transistors, and photovoltaics, on PPX thin films [22,23,24,25]. Lahann et al. reported the creation of patterned bioactive surfaces using biotin through click reactions with acetylene units at the surface of PPX films [26,27,28]. These papers highlight the defect-free nature of PPX films, which provide functionalized surfaces and excellent dielectric properties for developing these devices. While structurally modified PPX films have been prepared from various [2.2]paracyclophane derivatives using the Gorham process [29,30], the control of the molecular permeabilities of PPX thin films has not been explored, except in our previous study [15]. The surface polymerization of PPXs can provide a deposition approach for clean and defect-free nanometer thickness films with an effective nanofiltration performance, and the molecular separation property can be tuned to achieve free-volume sizes and specific interactions between their substituents and target solutes in PPXs. In this study, we also report the growth of metal–organic frameworks on imidazole-modified PPX nanofilms and investigate the resulting changes in the molecular permeabilities of composite membranes.

## 2. Materials and Methods

General: NMR spectra were recorded on a Bruker AVANCE 400 FT NMR spectrometer at 399.65 MHz and 100.62 MHz for ^1^H and ^13^C in CDCl_3_ solution. Chemical shifts are reported relative to internal tetramethylsilane. Mass spectra with electrospray ionization were obtained on a Bruker Daltonics micrOTOF II (Bruker, Billerica, MA, USA). Single-crystal structural analysis of 4-imidazole-[2,2]paracyclophane **1** was carried out using a Rigaku XtaLAB mini II (Rigaku, Tokyo, Japan), and the single crystal of **1** was prepared via recrystallization from toluene with slow evaporation of n-hexane. Surface morphologies of nanofilms were characterized using atomic force microscopy (AFM) images in non-contact mode with a JEOL JSPM-5400 system (JEOL, Tokyo, Japan) and field-emission scanning electron microscope (FE-SEM) images recorded in a Hitachi S-5000 FE-SEM (Hitachi, Tokyo, Japan) with Pt sputter coating. The XRD patterns of PPX film **2** were analyzed using wide-angle X-ray diffraction (Rigaku XRD-DSC (Rigaku, Tokyo, Japan)) with Cu K*α* radiation. The chemical compositions of films were determined using X-ray photoelectron spectroscopy (Kratos Analytical Axis Ultra (Shimazu, Kyoto, Japan)). Surface zeta potentials were determined via electrophoretic light scattering (Malvern Zetasizer Nano (Malvern Panalytical, Malvern, UK) using Al_2_O_3_ nanoparticles in various pHs. The thicknesses of **2** deposited on Si wafers were analyzed with a thin-film measurement system (Filmetrics F20-UV (Filmetrics, San Diego, CA, USA). 

All chemicals were purchased from commercial supplies and used without further purification. 

Synthesis of 1 [31]: The mixture of 4-amino-[2,2]paracycrophane (500 mg, 2.24 mmol), NH_4_Cl (300 mg, 5.60 mmol), and paraformaldehyde (168 mg) in a mixed solvent of 1,4-dioxane (9.0 mL) and water (3.0 mL) was stirred at room temperature for 15 min. Glyoxal (0.64 mL, 5.60 mmol) and one drop of H_3_PO_4_ were added to the reaction mixture, and the mixture was heated at 80 °C with stirring for 18 h. After cooling, 3.0 M NaOH aqueous solution (15.0 mL) was added, and the mixture was extracted with CH_2_Cl_2_. The organic layer was dried with MgSO_4_ and filtrated. After the evaporation of organic solvents, the crude product was purified via activated alumina column chromatography (eluent: CH_2_Cl_2_ to CH_2_Cl_2_: ethyl acetate = 1:1 *v*/*v*) and sublimated under vacuum to give a white solid. Yield 0.38 g (62%). ^1^H-NMR (400.52 MHz, CDCl_3_) δ 7.75 (s, 1H), 7.25 (s, 1H), 7.20 (t, J = 1.1 Hz, 1H), 6.56–6.70 (m, 5H), 6.52 (dd, J = 8.0, 1.8 Hz, 1H), 6.41–6.44 (m, 1H), 2.87–3.28 (m, 7H), 2.66–2.74 (m, 1H).^13^C-NMR (100.13 MHz, CDCl_3_) δ 142.10, 139.60, 139.53, 136.98, 136.87, 136.67, 133.58, 133.12, 133.02, 132.83, 132.02, 130.05, 129.44, 127.16, 119.71, 35.46, 35.05, 34.78, 32.66. FT-IR: 3045, 3022,1556 cm^−1^ (imidazole). ESI-TOF HRMS (APCI): *m*/*z* 275.1538 (calcd. *m*/*z* 275.1543 for C_19_H_18_N_2_ [M]^+^). 

Coating process of PPX films 2: The coatings of **2** on Si wafers and UF membranes (Biomax polyethersulfone (normal molecular weight limit: 50 kDa, diameter: 25 mm)) were carried out via a CVD polymerization process starting from **1** using an SCS Labcoter 2 parylene deposition system (Specialty Coating Systems). The monomer **1** was first sublimated at 175 °C, and the sublimated species were then introduced into the pyrolysis zone, which was heated at 650 °C. The *p*-quinodimethane derivatives generated during the pyrolysis process were polymerized onto target substrates, which were placed on a rotating holder at room temperature. The pressure inside the chamber was maintained at 4.0 Pa throughout the CVD process. The thicknesses of the resulting coatings of **2** on the target substrates were determined by analyzing the light reflection from Si wafer samples coated with **2** using F20-UV. 

Preparation of 2/ZIF-8 nanocomposite membrane: **2**/ZIF-L composite films were prepared via sequential permeation of UF membranes coated with **2** in aqueous solutions of Zn(NO_3_)_2_∙6H_2_O (25 mM, 10 mL) and 2mIM (170 mM, 10 mL) at a pressure difference of 0.4 MPa with two intermediate washings with DI water (10 mL) using a dead-end filtration system. 

Nanofiltration: Nanofiltration experiments were performed in five repeats in a dead-end stirred cell (Merck Millipore, Model 8010 (Merck KGaA, Darmstadt, Germany) at 20 °C and 0–0.5 MPa. Data on water permeance and dye rejection performance were the average value using five membranes coated with **2** or **2**/ZIF-L, fabricated using five independent processes under the same conditions. The measurements were performed after the initial compaction phase until permeance reached a steady state. The effective membrane area was 4.1 cm^2^, and the stirring speed was 500 r.p.m. Permeate samples for solvent permeance measurements were collected at intervals of 10 min, and samples for rejection evaluations were taken after 2 h. The solvent permeance *J* (L m^−2^ h^−1^ MPa^−1^) was determined using the equation *J* = *V*/(*A* × *t* × *p*), where *V* is the permeate volume, *A* is the effective membrane area, *t* is the unit time, and *p* is the applied pressure. The molecular weight cut-off (MWCO) of the membrane was tested by filtrating a series of polyethylene glycols (PEGs) with an average molecular weight of 330, 601, 1490, 3060, 6550, 12,200, 25,800, and 41,300 Da with a concentration of 150 ppm at 0.4 MPa. The MWCO of the membrane was defined as the molecular weight of a PEG, which has 90% rejection rate, and rejection rates were determined from the average values for three samples. 

## 3. Results and Discussion

**Scheme 1:**
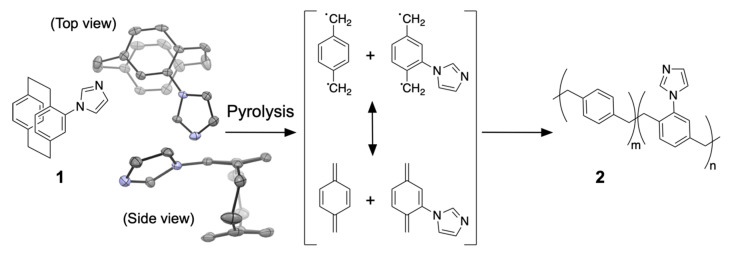
Polymerization of 4-imidazole-[2,2]paracyclophane **1** via the CVD process and single-crystal X-ray structures of **1** (displacement ellipsoids are shown at the 50% probability level and hydrogen atoms omitted).

Imidazoles are important conjugated heterocyclic components in functional molecules and have been used in various applications, including drugs and ionic liquids [32,33]. In this study, an imidazole-substituted derivative **1** was synthesized from racemic 4-amino[2.2]paracyclophane, which serves as a monomer for PPX film **2** (Scheme 1) [31,34]. Compound **1** was purified via column chromatography and vacuum sublimation. A single-crystal X-ray structure of **1** (CCDC2109296) revealed a distortion of the [2.2]paracyclophane skeleton (Scheme 1 and Figure S1). Due to the steric repulsion, the carbon–carbon distance for the methylene bridge near the imidazole unit was longer than the other bridge. Differential scanning calorimetry (DSC) analysis of **1** exhibited a single endothermic peak at 156 °C, corresponding to the melting point from a crystal to a liquid state (Figure S2). The melting point of **1** was lower than that of [2.2]paracyclophane without a substituent (melting point: 286 °C), indicating that the introduction of imidazole and the distortion of the cyclic skeleton reduce intermolecular interaction among **1** in the crystal. Thermogravimetric analysis (TGA) of **1** under a N_2_ atmosphere revealed that a 10% weight loss occurred at 242 °C (Figure S3). These thermal properties of **1** were utilized to determine CVD deposition conditions, with the sublimation of **1** performed at 175 °C under vacuum conditions.

**Figure 1:**
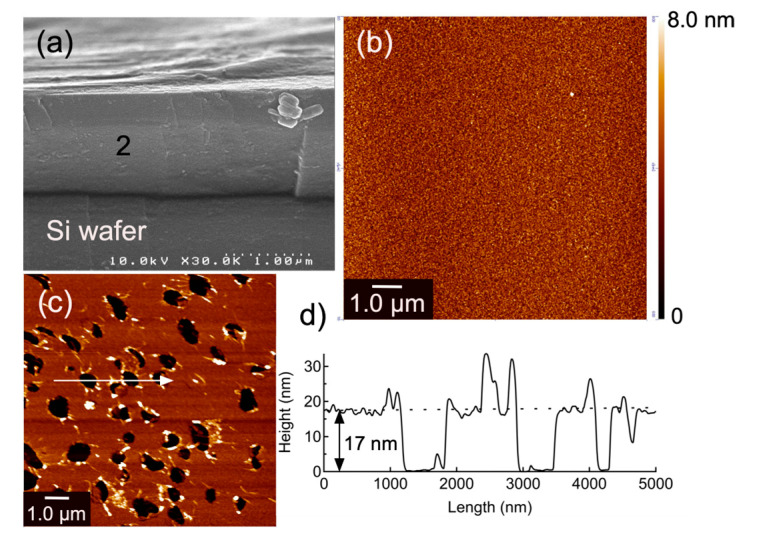
(**a**) FE-SEM image and (**b**) AFM image (R_a_ = 0.97 nm) of **2** films (thickness: 1.0 mm) deposited onto a Si wafer. (**c**) AFM image of **2** film damaged by 5 min of sonication in water. (**d**) Line scan of height change at the arrow shown in (**c**).

A *p*-Quinodimethane derivative formed through pyrolytic cleavage of vapored **1** under vacuum spontaneously polymerized into **2** at the surface of a Si wafer within the chamber (Scheme 1). Figure 1a,b show images of field-emission scanning electron microscopy (FE-SEM) and atomic force microscopy (AFM) of **2** on a Si wafer. Polymer **2** is insoluble in all common solvents. The FE-SEM image of the cross-section sample indicates the deposition of polymer film **2** with a 1.0 mm thickness on a Si wafer. The AFM image of **2** revealed a smooth and uniform surface composed of nanometer-sized particle assemblies (Figure S4), and its height variations were less than 1.0 nm. The film thickness of **2** can be controlled within 10.0 nm to 1.0 mm by changing the initial amount of **1** for the CVD process. The film thicknesses of **2** of less than 100 nm were determined from the AFM image analysis of damaged samples via ultrasonication in water (Figure 1c). Circular domains appeared after ultrasonication, and the film thicknesses of **2** were determined from the average differences between the two levels in the AFM images. The thicknesses from the AFM analyses agreed with the values from the optical measurement. 

**Figure 2:**
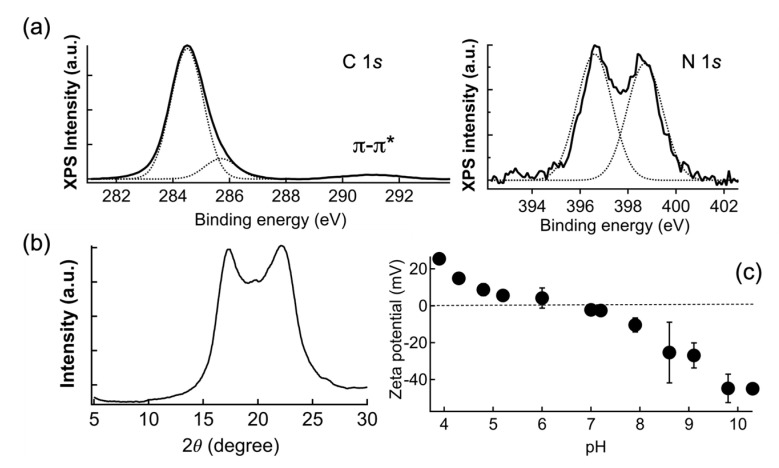
(**a**) C 1*s* and N 1*s* XPS spectra (dotted line: deconvolution for general chemical species identification) of **2** deposited on a Si wafer. (**b**) XRD pattern and (**c**) effect of pH on surface zeta potentials of **2** films.

We investigated the chemical composition of the **2** films via X-ray photoelectron spectroscopy (XPS). The XPS survey spectra revealed that **2** films contained 86.5 at. % carbon, 6.4 at. % nitrogen, and 7.1 at. % oxygen (Figure S5). The carbon/nitrogen ratio in the XPS of **2** almost agreed with the theoretical ratio (nominal values; 89.1 at. % carbon and 10.9 at. % nitrogen), and the presence of oxygen can be ascribed to the adsorption of oxygen at the surface of **2** after the deposition process. The C 1*s* were deconvoluted into two peaks positioned at 284.5 and 285.8 eV, which can be assigned to aliphatic and aromatic carbons (C=C/C-C) and carbon atoms bonded to N (C-N), respectively (Figure 2a) [35]. For N 1*s*, two characteristic peaks at 396.6 and 398.7 eV can be attributed to amine and imine nitrogen in imidazole units in **2** [36]. Consequently, the XPS spectra agree with the expected chemical structure of **2**. 

The polymerized film **2** showed a melting point of 284 °C as determined via DSC (Figures S6 and S7). The melting point of **2** was compatible with the reported value for Parylene C (290 °C). The wide-angle X-ray diffraction (XRD) pattern of 1.0 mm thick film **2** peeled off from the substrate showed two sharp peaks at 2*q* = 17.6 and 22.5° (Figure 2b) [37]. Out-of-plane XRD of an 11.1 nm thick film **2** on the Si wafer also displayed two peaks at 2*q* = 16.8 and 21.1°, indicating that the crystalline structure of **2** was not altered by reducing the film thickness (Figure S8). The film **2** deposited under the standard CVD condition was a semi-crystalline material and contained two *α* and *β* phases corresponding to monoclinic and hexagonal orientations of polymer backbones [38,39].

The hydrophobicity/hydrophilicity property of surfaces was analyzed considering the contact angle of a water droplet. The water droplet contact angle for **2** deposited on a Si wafer (76.2 ± 1.2°) was lower than that of PPX lacking any substituents (84.8 ± 1.3°) [40], suggesting that the introduction of polar imidazole side chains into PPX backbones slightly improved surface hydrophilicity. Figure 2c shows the surface zeta potential versus pH for **2** at room temperature. The surface of **2** exhibited positive and negative charges at low and high pH with an isoelectronic point around pH 7.0, indicating amphoteric characteristics of imidazole units in **2** due to the formation of the imidazolium cation and imidazoline anion at low and high pH ranges [41,42,43,44]. After the **2**-coated Si wafer was immersed into 0.1 mol/L HCl aqueous solution for 8 h., the AFM topological image after the treatment with HCl showed no changes for the as-deposited **2** films (Figure S9). 

The **2** films were deposited onto ultrafiltration (UF) membranes (molecular cut-off of 50 kDa) via the CVD of **1**. The cross-cut SEM image of the UF membrane coated with **2** displays a uniform covering of the supporting membrane surface (Figure S10). The UF membranes coated with **2** (28.7 ± 1.2 nm thick) exhibited a water permeation flux of 9.4 ± 0.35 L m^−2^ h^−1^ at a pressure difference of 0.5 MPa (Table 1). The water flux of **2**-coated membranes increased linearly with an increasing pressure difference in the 0.1–0.5 MPa range and was inversely proportional to the film thickness (Figure S11). The MWCO of **2** was 7.0 kDa, as determined through a solute filtration experiment with neutral PEGs with various molecular weights (Figure S12) [45]. 

The nanofiltration performance of **2** was evaluated by rejecting four types of water-soluble dyes with different molecular sizes, molecular weights, and charges (Table 1). The concentration of feed dye solutions was 10 mM, and the concentrations of residual dye in filtrates were determined by the changes in the absorbance in the visible absorption spectra. When a colored aqueous solution containing negatively charged Rose Bengal (RB) (Mw 1017.64 g/mol, Figure S13) was filtered in dead-end mode using the **2**-coated membranes, **2** could altogether reject RB with a molecular width of 1.2 nm, and the filtrate was a colorless liquid. This observation suggests the CVD-deposited **2** on the supported UF membranes had no defects. Dye rejection rates for methyl orange (MO) (Mw 327.33 g/mol), Rhodamine 6G (RH) (Mw 479.02 g/mol), and methylene blue (MB) (Mw 319.85 g/mol) were also examined at pH7.0, and they were 75 ± 3%, 60 ± 5%, and 26 ± 3%, respectively. Despite a slight difference in molecular sizes between MO and MB, **2** should not hinder the permeation of positively charged MB at pH 7.0. In contrast, the rejection of MB increased to 44 ± 2% at pH 5.0. Thus, **2** films can change the diffusivity of negatively charged dyes in response to the protonation degree of imidazole units [41,42,43,44]. 

**Figure 3:**
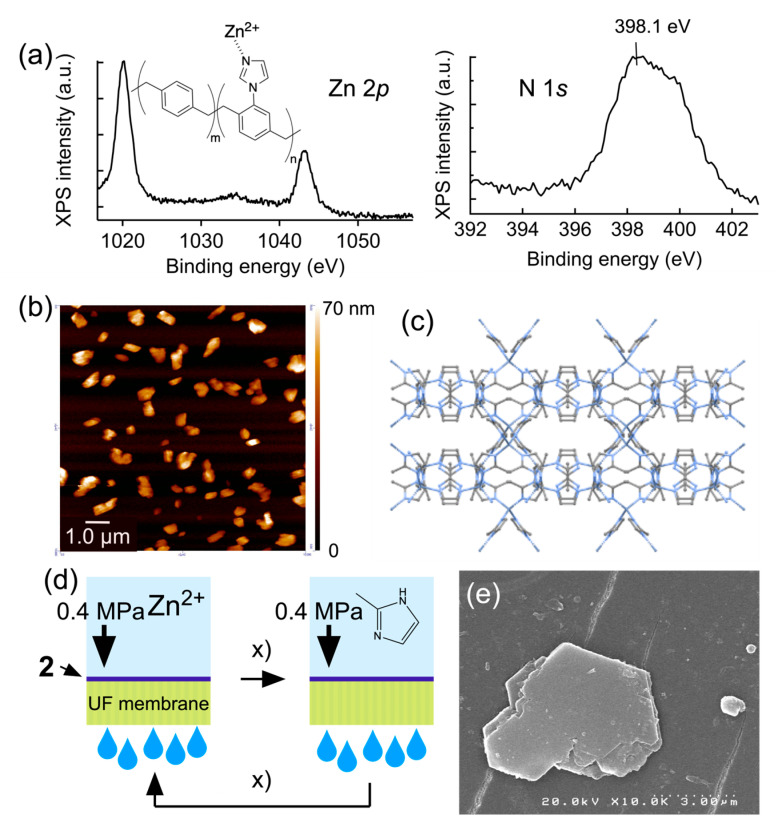
(**a**) Zn 2*p* and N 1*s* XPS spectra of **2** film after treatment with an aqueous solution of Zn(NO_3_)_2_∙6H_2_O for 5 min. (**b**) AFM image of the surface of a Si wafer coated with **2** after alternative immersing of two aqueous solutions of 2mIM and Zn^2+^. (**c**) The structure of ZIF-L (blue: nitrogen, black: carbon). (**d**) Growth process of ZIF-L on **2** deposited onto UF membrane (x: washing process with water). (**e**) FE-SEM image of ZIF-L crystal grown on **2** deposited onto UF membrane.

Zeolitic imidazolate frameworks (ZIFs) have been constructed by connecting tetrahedrally coordinated metal ions with imidazolate linkers [46,47,48]. ZIFs, composed of Zn^2+^ ions and 2-methyl imidazole (2mIM), have been widely investigated as durable porous materials in membranes for molecular separation processes [49,50,51]. In this study, we expected an improvement in the filtration performance of **2**/ZIF nanocomposite membranes due to the direct growth of ZIF crystals on the surface of **2** thin films. First, the **2**-coated Si wafer was immersed into an aqueous solution of Zn(NO_3_)_2_∙6H_2_O for 5 min, and the treated substrates were washed with water two times (Figure S14). As shown in Figure 3a, the XPS spectrum of Zn 2*p* revealed the binding energies of Zn 2*p*_3/2_ at 1020.2 eV and Zn 2*p*_1/2_ at 1043.2 eV, confirming the presence of Zn^2+^ in **2** (Figure S15) [52]. Moreover, the peaks of N 1*s* shifted to higher binding energies due to coordination with Zn^2+^ with imidazole units in **2**. XPS analyses revealed that Zn^2+^ ions could accumulate in **2** films by forming coordination bonds with imidazole units. 

Metal–organic frameworks were grown on the surface of **2** by alternately immersing two aqueous solutions of 2mIM and Zn^2+^ with a washing step [53]. The height of domains in the AFM images increased when the number of process cycles was increased (Figure 3b and Figure S16). The XRD peak positions of the composite film are almost consistent with the reported values of ZIF-L (Figure S17) [54]. ZIF-L is composed of two-dimensional layers with a cushion-shaped cavity of 9.4 × 7.0 × 5.3 Å (Figure 3c). This layered structure is stabilized by the intertwining layers of the monodentate Hmim and free Hmim molecule through hydrogen bonding. The water droplet contact angle of **2** modified with ZIF-L crystals (121.2 ± 4.2°) was higher than that of **2** due to the formation of hydrophobic ZIF-L crystals on the surface of **2** and the increase in surface roughness [55]. 

The ZIF-L crystals were formed on the surface of **2** (21.2 ± 1.5 nm thick) on UF membranes through alternative permeation of Zn^2+^ and 2mIM aqueous solutions at a pressure difference of 0.4 MPa (Figure 3d). The FE-SEM image of the **2**/ZIF-L nanocomposite membrane shows a thin flaky crystal on the surface of **2** (Figure 3e). The composite membrane performed better in rejecting charged dyes than **2** alone (Table 1). The rejection of MB molecules (17.0 × 7.6 × 3.3 nm) was significantly improved from 26% to 52% due to the adsorption into the internal cavity of ZIF-L. The rejection of MB gradually decreased when the passing volume of an aqueous solution of MB was increased, maintaining the water flux (Figure S18). 

## 4. Conclusions

To summarize, we have demonstrated the nanofiltration performance of PPX **2** thin films possessing imidazole side chains prepared on the surface of commercial UF membranes using a dry CVD process. The uniform and stable **2** PPX films with a few tens of nanometers were formed via the surface polymerization of imidazole-substituted monomers generated by the gas-phase pyrolysis of [2.2]paracyclophane derivatives without the use of solvents and catalysts. The resulting **2** films showed an increase in rejection rates for the cationic dyes RH and MB due to the protonation of imidazole units in **2** below pH 7. Furthermore, the imidazole units in **2** could accumulate Zn^2+^ ions through the coordination bond, and ZIF-L architectures were constructed on **2**. The **2**/ZIF-L nanocomposite membranes displayed improved dye rejection rates for water-soluble organic dyes with increasing water flux compared to **2**. The clean and robust PPX films presented here provide a platform for molecular separations through tuning free-volume sizes and specific interactions between their substituents and target solutes in PPXs. Further studies are underway to establish the potential for various applications, such as an effective separation of biomolecules in biomedical devices and ultrathin ion-exchange membranes of energy storage devices.

## Figures and Tables

**Table 1:** Water fluxes and dye rejection ratios of **2** and **2**/ZIF-L composite membranes.

	Water Flux	Dye Rejection ^a^ (%)			
	(L m^−2^ h^−1^ MPa^−1^)	RB ^b^	MO ^c^	RH ^d^	MB ^e^
**2**	19.8 ± 0.7	95 ± 2	75 ± 3	60 ± 5	26 ± 3
**2**/ZIF-L	19.2 ± 1.0	98 ± 2	90 ± 6	75 ± 3	52 ± 2

^a^ (Dye) = 10 mM, ^b^ RB: Rose Bengal, ^c^ MO: methyl orange, ^d^ RH: Rhodamine 6G, ^e^ MB: methylene blue.

## Data Availability

Data is contained within the article or Supplementary Material.

## Supplementary Materials

The following supporting information can be downloaded at: https://www.mdpi.com/article/10.3390/polym15153309/s1. Figure S1. Angle difference of two ethylene bridges in **1**; Figure S2. DSC profile of **1** during the heating process. Figure S3. TGA profile of **1** under N_2_ atmosphere. Figure S4. AFM image (2 × 2 mm) of **2** on a Si wafer. Figure S5. XPS wide-scan spectrum of **2** deposited onto a Si wafer. Figure S6. DSC profile of **2** during the heating process. Figure S7. TGA profile of **2** under N_2_ atmosphere. Figure S8. Out-of-plane XRD of 11.1 nm thick film **2** on the Si wafer. Figure S9. AFM image of **2** after treatment with 0.1 mol/L HCl. Figure S10. Cross-cut FE-SEM image of **2**-coated UF membrane; (a) cross-cut image of **2**-coated polyethersulfone layer in UF membrane and (b) magnified image of the surface of the **2**-coated membrane. Figure S11. Dependences of water flux of **2** on pressure (a) and film thickness (b) when using a dead-end membrane filtration system at room temperature. Figure S12. Determination of MWCO for **2** from the rejection curve of PEGs. Figure S13. Chemical structures of water-soluble dyes. Figure S14. AFM image of **2** film after treatment with an aqueous solution of Zn(NO_3_)_2_∙6H_2_O. Figure S15. XPS wide-scan spectrum of **2** with an aqueous solution of Zn(NO_3_)_2_∙6H_2_O. Figure S16. Height change with an increasing number of cycles for the growth of ZIF-L on **2**. Figure S17. XRD patterns of **2**/ZIF-L composite film on a Si wafer and reference patterns of ZIF-8 and ZIF-L. Figure S18. Change in rejection ratio of **2**/ZIF-L composite membrane during continuous feeding of an aqueous solution of MB.
