# Supplementary material for: Nanofiltration Performance of Poly(p-xylylene) Nanofilms with Imidazole Side Chains
Source: Polymers (Basel). 2023 Aug 4;15(15):3309. doi: 10.3390/polym15153309 (PMC10422224; doi:10.3390/polym15153309)
Supplement: Supplementary file 1 [file polymers-15-03309-s001.zip › polymers-2497112-supplementary.pdf]

Nanofiltration Performance of Poly(p-xylylene) Nanofilms with Imidazole Side Chains

Satsuki Yoshida, Takeshi Shii, Yu Kitazawa, Manuela L. Kim, Eugenio H. Otal, Yoshiyuki Hattori and Mutsumi Kimura *

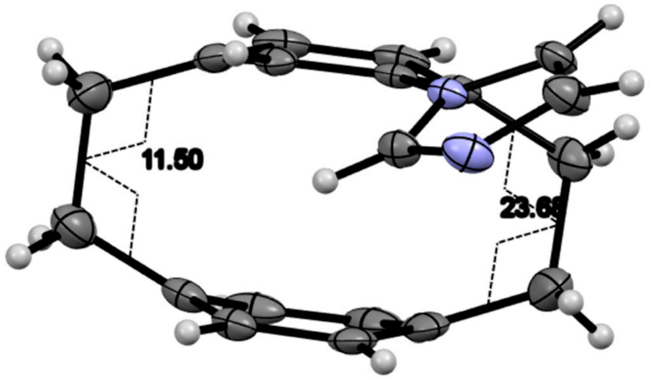

Figure S1 Angle difference of two ethylene bridges in **1**

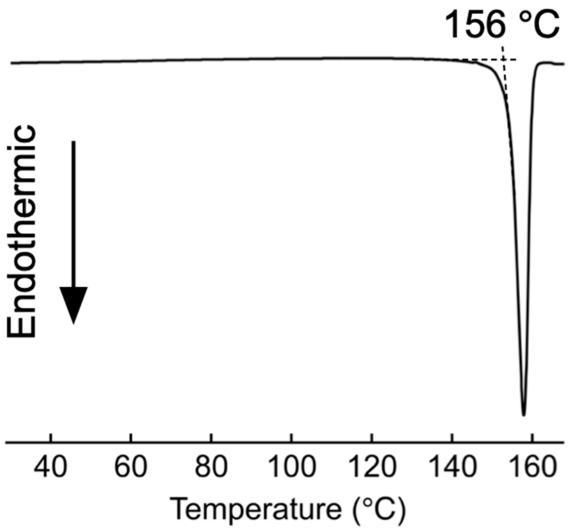

Figure S2 DSC profile of **1** at the heating process (heating rate: 10 °C/min).

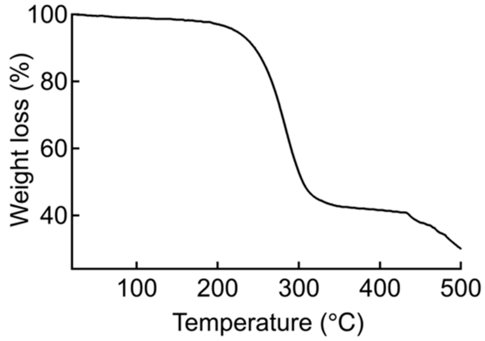

Figure S3 TGA profile of **1** under N_2 atmosphere (heating rate: 10 °C/min).

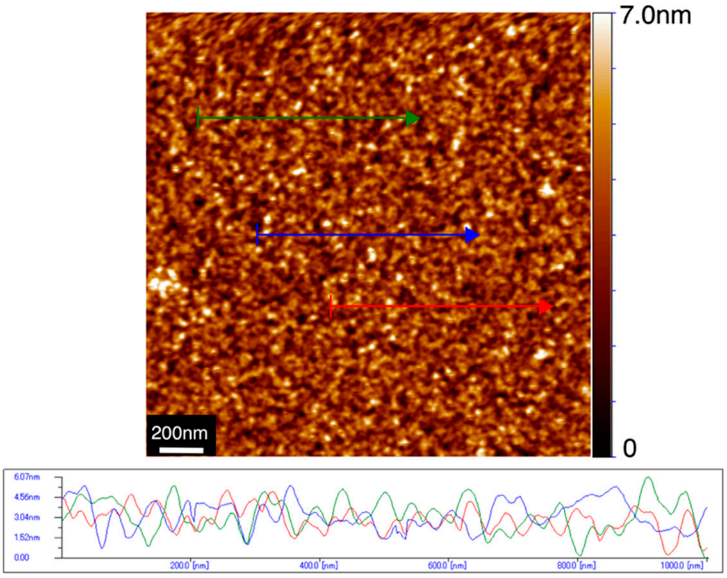

Figure S4 AFM image (2x2 μm) of **2** on a Si wafer (R_a = 0.87 nm) and line scans of height changes at three arrows (red, blue, and green arrows).

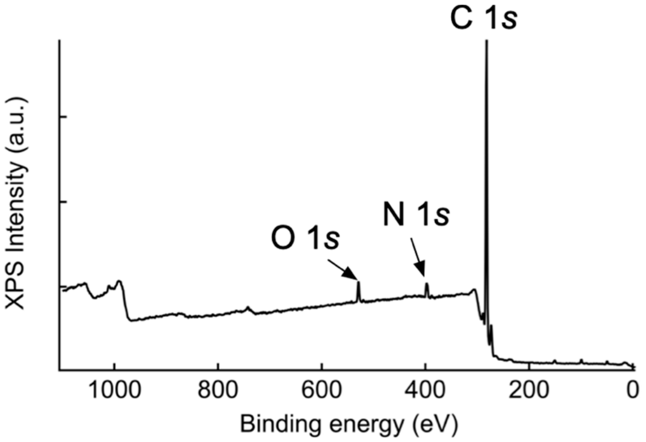

Figure S5 XPS wide-scan spectrum of **2** deposited onto a Si wafer.

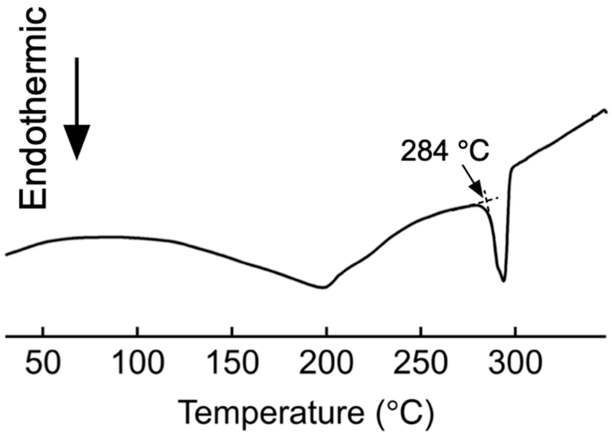

Figure S6 DSC profile of **2** at the heating process (heating rate: 10 °C/min).

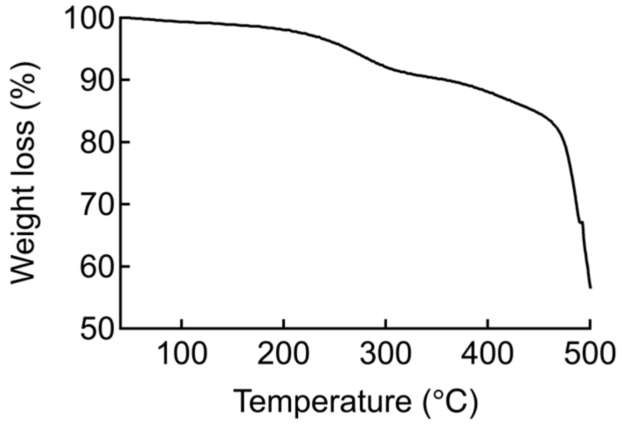

Figure S7 TGA profile of **2** under N_2 atmosphere (heating rate: 10 °C/min).

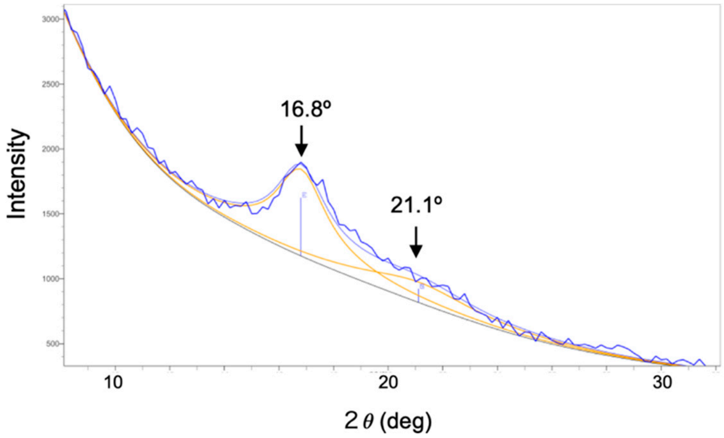

Figure S8 Out-of-plain XRD of 11.1 nm-thick film **2** on the Si wafer (blue line: XRD reflection, yellow line: deconvolution into two peaks, and gray line: base line).

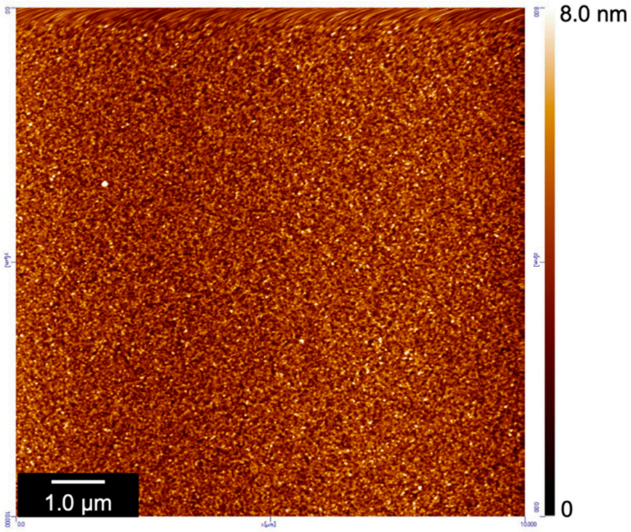

Figure S9 AFM image of **2** after treatment with 0.1 mol/L HCl.

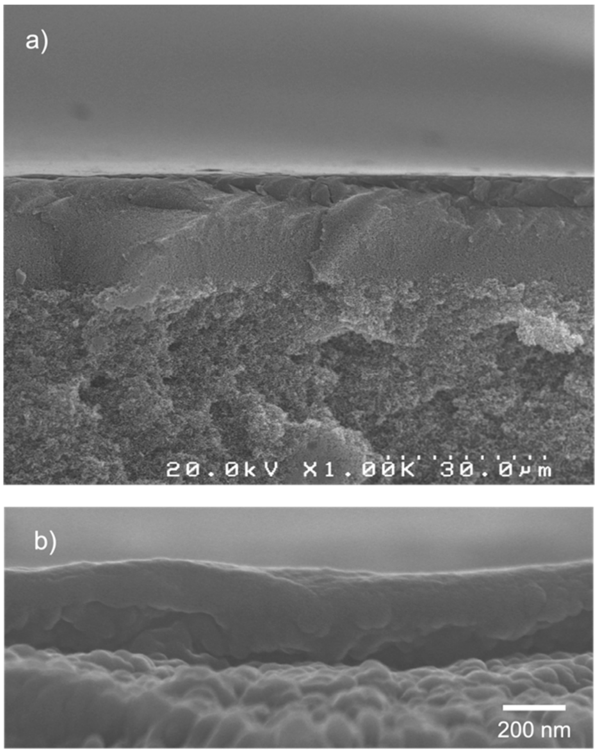

Figure S10 Cross-cut FE-SEM image of **2**-coated UF membrane;

a) Cross-cut image of **2**-coated polyethersulfone layer in UF membrane and

b) Magnified image for the surface of the **2**-coated membrane.

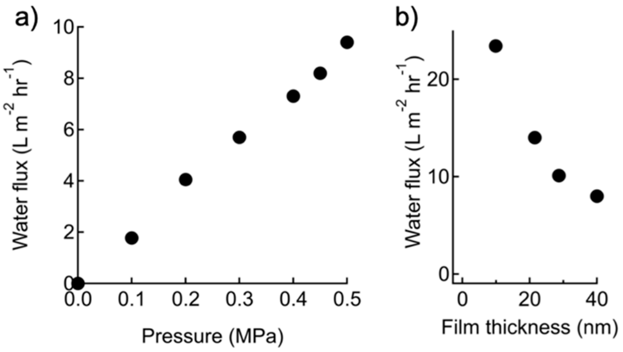

Figure S11 Dependences of water flux of **2** on pressure (a) and film thickness (b) by using a dead-end membrane filtration system at room temperature.

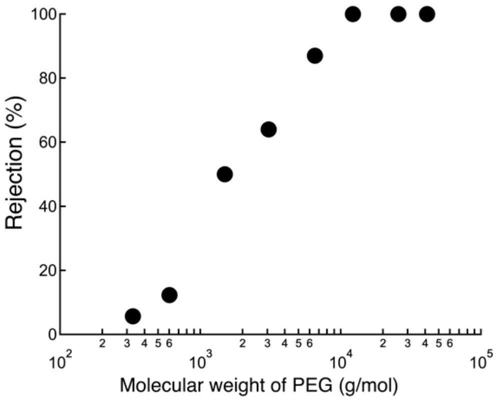

Figure S12 Determination of MWCO for **2** from the rejection curve of PEGs.

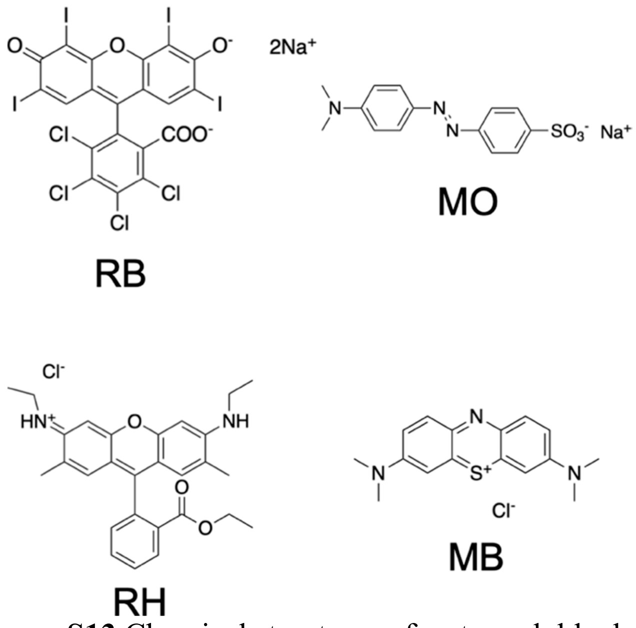

Figure S13 Chemical structures of water-soluble dyes.

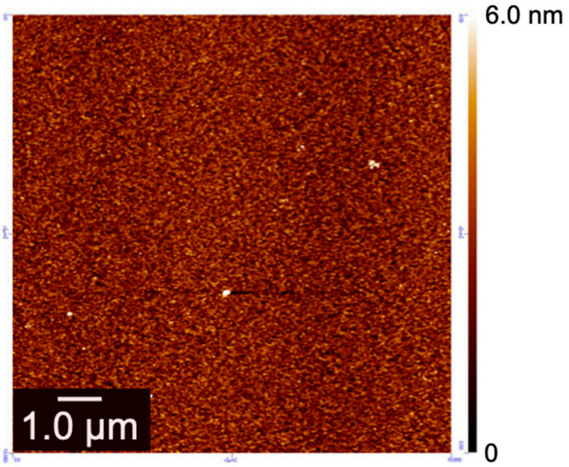

Figure S14 AFM image of **2** film after treatment with an aqueous solution of $Zn(NO_3)_2 \cdot 6H_2O$.

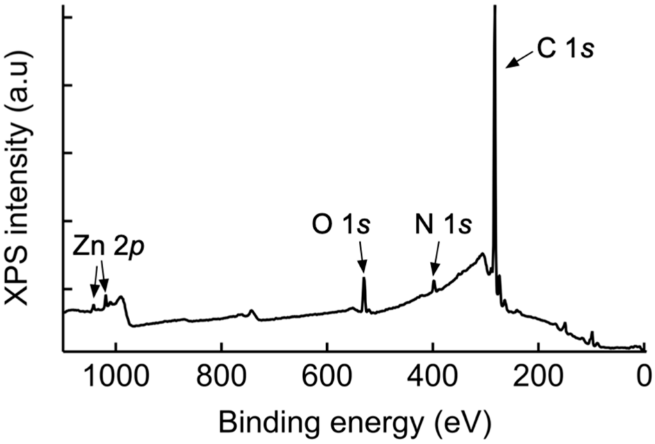

Figure S15 XPS wide-scan spectrum of **2** with an aqueous solution of $Zn(NO_3)_2 \cdot 6H_2O$.

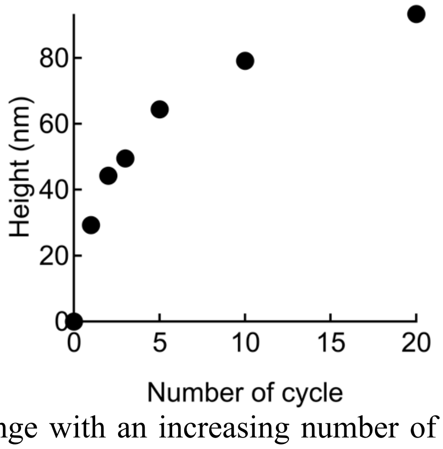

Figure S16 Height change with an increasing number of cycles for the growth of ZIF-L on **2**. Heights (average values for three samples) were determined by AFM.

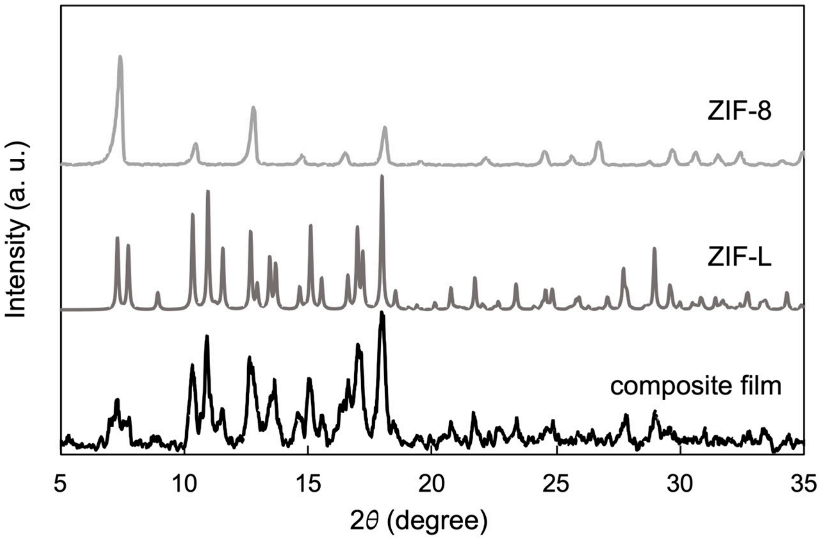

Figure 17 XRD patterns of **2**/ZIF-L composite film on a Si wafer and reference patterns of ZIF-8 and ZIF-L.

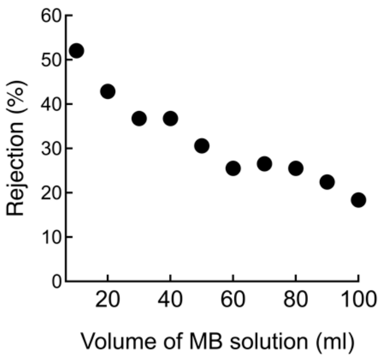

Figure S18 Rejection ratio change of **2**/ZIF-L composite membrane during continuous feeding of an aqueous solution of MB ([MB] = 10 μM).
